# Effects of age and sex on epigenetic modification induced by an acute physical exercise: Retraction

**DOI:** 10.1097/MD.0000000000009706

**Published:** 2018-01-19

The article, “Effects of age and sex on epigenetic modification induced by an acute physical exercise”,^[1]^ which appeared Volume 96, Issue 44 *Medicine*, has been retracted for plagiarism. A reader has brought to our attention that substantial parts of the study design and language have been found in the previously published study, “Exercise-conditioned plasma attenuates nuclear concentrations of DNA methyltransferase 3B in human peripheral blood mononuclear cells”,^[2]^ which appeared in Volume 3 of *Physiological Reports.*
